# HEALTH-RELATED QUALITY OF LIFE AND PARTICIPATION AFTER INPATIENT REHABILITATION OF SEPSIS SURVIVORS WITH SEVERE SEQUELAE: A COHORT STUDY

**DOI:** 10.2340/jrm.v56.18670

**Published:** 2024-07-02

**Authors:** Ulf BODECHTEL, Thea KOCH, Lars HEUBNER, Peter SPIETH, Ines RÖßLER, Jan MEHRHOLZ

**Affiliations:** 1 Klinik Bavaria in Kreischa, Kreischa; 2 Klinik für Anästhesiologie und Intensivtherapie, Medizinische Fakultät, Carl Gustav Carus, Technische Universität Dresden, Dresden; 3 Wissenschaftliches Institut, Private Europäische Medizinische Akademie der Klinik Bavaria in Kreischa, Kreischa; 4 Department of Public Health, Medizinische Fakultät, Carl Gustav Carus, Technische Universität Dresden, Germany

**Keywords:** quality of life, rehabilitation, sepsis, social participation

## Abstract

**Objective:**

To describe health-related quality of life and participation after rehabilitation of severely affected sepsis survivors.

**Design:**

Cohort study.

**Subjects/Patients:**

Patients with severe sequelae after sepsis treated in a multidisciplinary rehabilitation pathway were included.

**Methods:**

Patient characteristics at the time of diagnosis, and the outcome 3 months after discharge from rehabilitation are described. At that time, health-related quality of life, social participation, and the rate of living at home were measured.

**Results:**

Of the 498 patients enrolled, 100 severely impaired patients were transferred for a multidisciplinary rehabilitation approach. Fifty-five of them were followed up at 3 months. Descriptive and inference statistics showed that 69% were living at home with or without care. Health-related quality of life and participation scores were 0.64 ± 0.32 for the EQ-5D utility index and 54.98 ± 24.97 for the Reintegration of Normal Living Index. A multivariate regression model explaining health-related quality of life at 3 months included age, lower limb strength, and walking ability during rehabilitation (*r*^2^ = 0.5511). Participation at 3 months was explained by age, body mass index, lower limb strength, and duration of tracheal intubation (*r*^2^ = 0.6229).

**Conclusion:**

Patients who have experienced serious sepsis with severe sequelae can achieve a moderate level of quality of life and participation within a multidisciplinary pathway.

LAY ABSTRACT

Our study aimed to describe the health-related quality of life, participation, and the number of patients who were living at home after rehabilitation. Our cohort study included 100 patients who had severe impairment after sepsis and were transferred to our rehabilitation clinic. We could analyse 55 of these patients 3 months after discharge. Of these patients, 69% were living at home. The scores for health-related quality of life and participation were reasonable given the severity of the illness. The quality of life after 3 months could be attributed to factors such as the patient’s age, strength in the lower limbs, and walking ability during rehabilitation. Social participation after 3 months could be linked to variables such as the patient’s age, body mass index, lower limb strength, and the duration of tracheal intubation. In conclusion, severely affected sepsis survivors who undergo multidisciplinary rehabilitation can have moderate levels of quality of life and participation.

Sepsis is a life-threatening condition characterised by a dysregulated immune response to infection, leading to multiple organ dysfunction, and is associated with severe morbidity and mortality, making it a critical focus for research and intervention efforts (1, 2). Beyond the immediate threat to life, surviving sepsis can have profound long-term effects on patients’ health-related quality of life (HRQoL) and ability to participate in daily activities, requiring a comprehensive approach to their care and rehabilitation (3, 4).

The recovery process for sepsis survivors extends well beyond the acute phase of the illness, and many patients face ongoing challenges in resuming a normal life. Iwashyna et al. highlight the long-term cognitive impairment and functional disability that can persist in sepsis survivors, underscoring the importance of assessing and addressing HRQoL in this population (5). In addition to physical impairment, the ability to engage in social and occupational activities, also known as social participation, is an essential component of a patient’s overall well-being and reintegration into society. Limited participation can have profound social and economic consequences for individuals and their families, highlighting the importance of assessing and supporting participation outcomes in sepsis survivors (4). Given the short- and long-term consequences of sepsis with severe sequelae on physical function, quality of life, and social participation, the rehabilitation of these patients remains a major challenge in rehabilitation medicine. Recently, important research gaps after sepsis have been identified, including: (a) few studies of in-hospital or post-hospital interventions to improve longer-term survival and quality of life; and (b) limited data on how to identify patients most likely to benefit from interventions (6). Furthermore, there is increasing recognition of the need for multidisciplinary rehabilitation programmes to optimize patient outcomes, given the potential long-term impact of sepsis on HRQoL and participation (7). It is now well known that the development of tailored rehabilitation packages for critically ill patients is needed to improve functional outcomes and enhance HRQoL during the recovery process. On the one hand, specific rehabilitation contexts are thought to have a positive impact on the physical outcomes of sepsis survivors (8); on the other hand the best rehabilitation strategy after sepsis remains largely uncertain.

Few studies have examined the effects of a dedicated care pathway, from the acute phase through to rehabilitation, on the health-related quality of life and social participation of patients with severe sepsis (9).

Therefore, this study aims to describe the health-related quality of life and social participation of patients who had severe sepsis, 3 months after inpatient rehabilitation following a specific care pathway. We will also identify predictors of better health-related quality of life and social reintegration.

## METHODS

### Study design

We collected study data as part of a prospective and comprehensive cohort study, which aimed to describe the clinical characteristics and outcomes in critically ill septic patients, as previously reported (10).

We conducted this study accordingly to the Helsinki Declaration and with ethical approval by the local ethics commission (BO-EK-374072021) and registered the study before publication (German Register of Clinical Trials, DRKS00020495). We followed the STrengthening the Reporting of OBservational studies in Epidemiology (STROBE) guidelines to conduct this study and to write the manuscript.

### Patients and setting

From February 2020 to March 2023, all patients were recruited from various intensive care units (ICU) of the University hospital Dresden, Germany. After informed consent, patients with sepsis were enrolled in the Comprehensive Sepsis Center (CSC) Dresden – Kreischa study (10) and followed up for 3 months after discharge from rehabilitation. Patients were eligible if they were aged ≥ 18 years and diagnosed with sepsis or septic shock according to the International Consensus Definitions for Sepsis and Septic Shock (Sepsis-3) (10). Informed consent was obtained from the patient or a legal representative.

All patients in this cohort were eligible for our study if they were admitted for weaning and intensive rehabilitation to our post-ICU and rehabilitation clinic, and early rehabilitation departments at Klinik Bavaria Kreischa, Germany. Our post-acute unit integrates specialized approaches for sepsis survivors with severe sequelae due to critical illness polyneuropathy and myopathy (CIP/CIM) or septic encephalopathy, alone or in combination with other medical conditions like decompensated chronic or acute lung, heart, kidney, or liver diseases or possibly accompanying complex wounds. The customized rehabilitation and treatment programme, comprising weaning, mobilization, and rehabilitation, commences promptly following admission from acute intensive care hospitals. The rehabilitation pathway focuses on treating the main limitations caused by CIP/CIM (tetraparesis, neurogenic dysphagia, and respiratory failure due to neuromuscular weakness). The treatment of other medical consequences of sepsis, such as dialysis weaning or structured cannula removal, is also part of the patient pathway, as is proactive treatment planning, including ongoing assessment of treatment goals. Furthermore, the pathway includes an assessment and treatment plan for cognitive and mental impairment. Other challenges comprise treating recurrent infections and medical conditions such as complex wound care.

The rehabilitation programme involved physiotherapy and occupational therapy on weekdays, alongside other relevant therapies. Each patient received a personalised treatment plan based on their particular goals, such as regaining walking function and performing activities of daily living. A corresponding assessment is conducted regularly to monitor rehabilitation progress. However, the therapies’ content and intensity varied depending on the severity of the critical illness and the individual’s goals.

Sepsis patients who were not part of the CSC observational study at the University Hospital Dresden received the same rehabilitation pathway, but were excluded from our analysis.

### Measures and outcomes

We recorded patient characteristics, such as their age, sex, comorbidities, illness severity, and other clinical and demographic variables at the time of sepsis diagnosis. During the patients’ ICU stay, we collected baseline data using an electronic data management system that we had described earlier (10).

At the 3-month follow-up after discharge from rehabilitation, health-related quality of life (HRQoL) was assessed using the EQ-5D, a widely recognized instrument that measures health status across 5 dimensions (mobility, self-care, usual activities, pain/discomfort, and anxiety/depression) and provides a single summary index ranging from 0 (worst health) to 1 (perfect health) (11–13).

At this point we also measured social participation using the Reintegration of Normal Living Index (RNLI), a validated questionnaire that assesses the extent to which individuals are participating in their daily activities and social roles on a scale from 0 (no participation) to 100 (full participation) (13, 14). The RNLI consists of 11 declarative statements. The first 8 items represent “daily functioning”, while the remaining 3 items represent “perception of self”. The domains of the RNLI encompass indoor, community, and distance mobility, self-care, daily activities, recreational and social activities, family role(s), personal relationships, presentation of self to others, and general coping skills. The RNLI maximum score is 100 points.

All patients were contacted by letter and telephone by one of our experienced psychologists (I.R.) at the follow-up visit, as agreed in advance, and the EQ-5D and RNLI scores were also sent by letter.

Additionally, we assessed survival and the rate of living at home 3 months after discharge from rehabilitation, reflecting the proportion of patients who returned to their homes after hospital discharge with or without additional care.

### Statistical analyses

Descriptive and inference statistics were used dependent on type of data distribution. The global alpha level was set at 0.05. Frequencies and corresponding percentages were calculated and presented in relation to the initial number of patients in the study, e.g., the percentage of patients walking independently.

For the EQ-5D and RNLI scores, means and standard deviations were calculated to provide a measure of central tendency and variability.

To determine the factors associated with HRQoL and participation at 3 months after rehabilitation, after univariate analysis multivariate linear regression models were also applied. In our univariate analysis we searched for clinically important and significant predictors. Candidate variables included sex, age, height, weight, body mass index, Charlson Comorbidity Index, lower limb strength, upper limb strength, grip strength, walking ability, duration of tracheal intubation, duration of ventilation, and sequential organ failure assessment score at disease onset. These candidate variables were selected through discussion of their clinical meaning by the author team. In the next step we used multivariate regression models and included important predictors from the univariate analysis (*p*-value < 0.2). Predictor variables included lower limb strength, walking ability, upper limb strength, grip strength, duration of tracheal intubation, age, and body mass index. In the multivariate model selection process we systematically evaluated all possible combinations of predictors to identify the best-fitting model for each outcome measure (HRQoL and participation). We employed an algorithm called “best subsets approach” (implemented in PROC REG, SAS/STAT 9.4) with *r*^2^ selection method to determine the predictor subset that offered the best data fit based on statistical criteria, including adjusted *r*^2^ and Mallows’ Cp (to assess the multivariate regression model’s best fit). Mallows’ Cp was used to compare the fit of the regression models. To select the final multivariate regression model, we selected the model with the highest *r*^2^ and the lowest Mallows’ Cp. We used SAS/STAT 9.4 for all statistical procedures (SAS Institute Inc., Cary, NC, USA).

## RESULTS

**Fig. 1:**
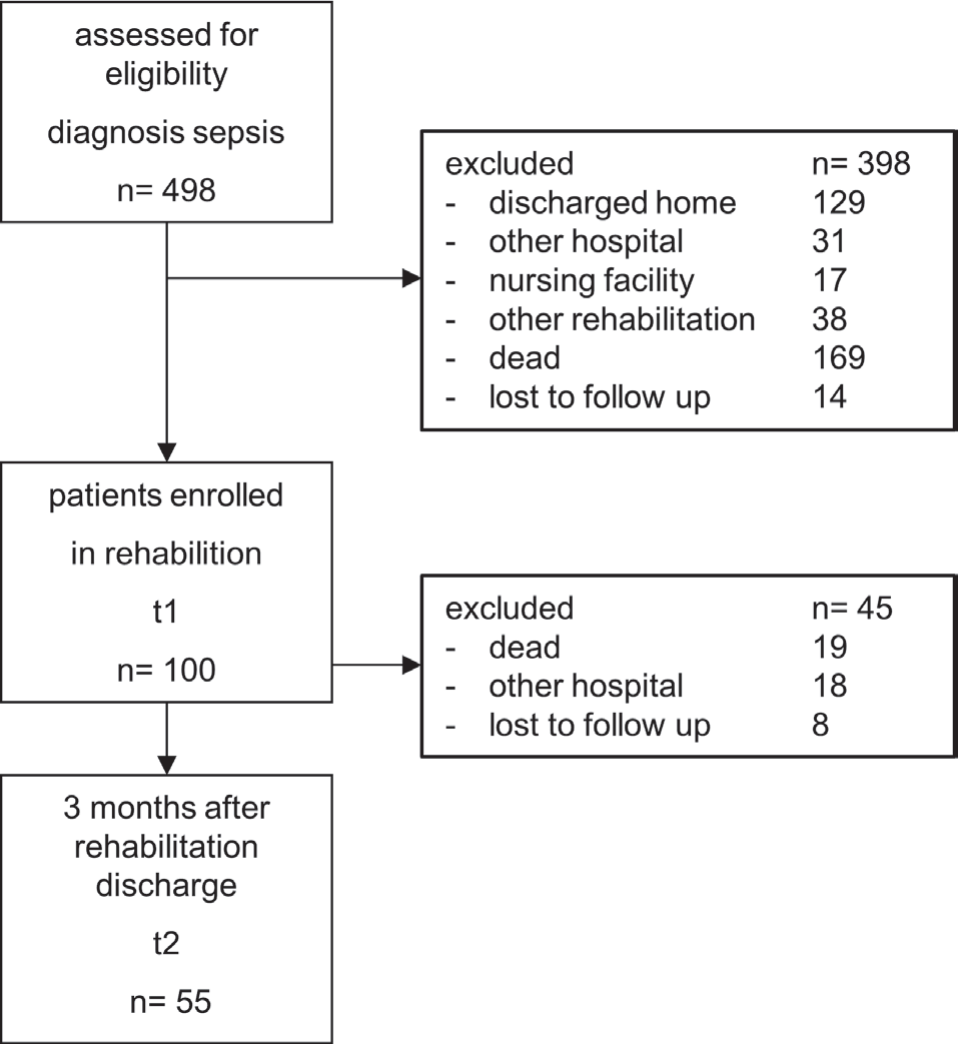
Flowchart.

We evaluated a total of 498 patients diagnosed with acute and severe sepsis between February 2020 and March 2023 in our cohort study (Fig. 1). Of the 498 patients enrolled, 100 patients with severe sequelae (20%) were transferred directly from ICU to our post-ICU and rehabilitation clinic. The mean length of stay was 31 ± 23 days, (median 27; interquartile range [IQR] 1–165) in the acute ICU and 119 ± 43 days (median 122; IQR 42–207) in the post-ICU and rehabilitation clinic. The mean duration of mechanical ventilation during rehabilitation was 17.98 ± 12.35 days. Tracheal intubation and renal replacement therapy were still present during rehabilitation and lasted 53.85 ± 56.19 days and 22.99 ± 18.53 days respectively. Nineteen out of 100 patients (19%) passed away during their stay in our post-ICU and rehabilitation clinic or rehabilitation unit.

Fifty-five patients were available for analysis at 3 months after discharge from rehabilitation (Fig. 1), and 38 (69%) of them lived at home with or without care. For these 55 patients the mean time from sepsis onset to follow up was 221 ± 66 days. Table I lists all demographic and clinical characteristics at study onset in our post-acute rehabilitation (T1).

**Table I:** Baseline characteristics at sepsis onset

Variable (*n* = 100)	Proportion
Age in years, mean (SD)	61.4 (11.3)
Female sex, *n* (%)	75 (75)
Body mass index, mean (SD)	30.2 (7.2)
Charlson Comorbidity Index, mean (SD)	3.7 (2.7)
Barthel Index pre-morbid, mean (SD)	79.3 (32.6)
Maximum Sepsis-related Organ failure Assessment score	11.4 (3.5)
Mechanical ventilation, *n* (%)	99 (99)
Tracheostomy tube, *n* (%)	72 (72)
Renal replacement therapy, *n* (%)	42 (42)
Wound requiring treatment, *n* (%)	37 (37)
Walking independently (FAC > 3), *n* (%)	0 (0)

Baseline was defined as the onset of sepsis. Based on this definition the duration of illness was defined as the time between the first day in ICU due to the onset of sepsis until the first admission to post-ICU acute rehab.

SD: standard deviation; FAC: functional ambulation categories.

Further clinical characteristics at study onset in our post-acute rehabilitation (T1) compared with 3 months’ follow-up are shown in Table II.

**Table II:** Patient characteristics on admission for rehabilitation and at 3 months’ follow-up

Factor	On admission to rehabilitation (*n* = 55)	At 3-month follow-up (*n* = 55)	Absolute risk difference	Needed to treat* *n*
Mechanical ventilation, *n* (%)	32 (58%)	1 (2%)	0.56	2
Tracheostomy tube, *n* (%)	40 (72%)	5 (9%)	0.63	2
Renal replacement therapy, *n* (%)	11 (29%)	5 (9%)	0.2	5
Wound requiring treatment, *n* (%)	4 (37%)	10 (18%)	0.19	6
Walking independently (FAC > 3), *n* (%)	0 (0%)	33 (60%)	0.6	4

Only patients who were available at the 3-month follow-up are displayed in the second column, which accounts for 55 of the initially included 100 patients. For these 55 patients the mean time from sepsis onset to follow up was 221 ± 66 days.

The absolute risk difference compares the incidence rate on admission with the rate at 3-month follow-up. The number needed to treat is equal to 1 divided by the absolute risk difference.

FAC: functional ambulation categories.

* The number needed to treat was rounded up.

### Health-related quality of life (EQ-5D)

The EQ-5D health-related quality of life mean utility score was 0.64 ± 0.32.

In the EQ-5D dimensions the following rates of extreme difficulties or problems were reported:

- in the mobility dimension 18% rated “I am confined to bed”;
- in the self-care dimension 16% rated “I am unable to wash or dress myself”;
- in the usual activities dimension 20% rated “I am unable to do my usual activities”;
- in the pain/discomfort dimension 6% rated “I have extreme pain or discomfort”;
- in the anxiety/depression dimension 6% rated “I am extremely anxious or depressed”.

After univariate regression analysis, we retained our significant predictor variables in the multivariate analysis and selected the final multivariate regression model using the best subset approach (see Table III). Our final multivariate regression model explaining health-related quality of life at 3 months included age, lower limb strength, and walking ability at rehabilitation (*r*^2^ = 0.5511; Mallows’ Cp = 0.9840). Therefore, younger patients with better leg strength and early walking ability had higher health-related quality of life at 3 months after rehabilitation.

**Table III:** Results of the regression analyses for health-related quality of life and social participation

Predictor variable	R^2^	Cp	Regression coefficient	*p*-value
Health-related quality of life				
Univariate regression				
Lower limb strength	0.361	–0.476	0.015	0.005
Walking ability	0.223	1.352	0.086	0.032
Upper limb strength	0.191	1.788	0.012	0.005
Grip strength	0.146	2.384	0.019	0.056
Duration of tracheal intubation	0.021	4.052	–0.006	0.134
Age	0.008	4.22	–0.008	0.054
Body mass index	0.002	4.3	–0.001	0.187
Multivariate regression (combinations of 2 variables)				
Predictor variable combination	R^2^	Cp		
Lower limb strength & age	0.492	–0.223		
Lower limb strength & walking ability	0.425	0.669		
Lower limb strength & duration of tracheal intubation	0.389	1.14		
Multivariate regression (combinations of 3 variables)				
Predictor variable combination	R^2^	Cp		
Lower limb strength & age & walking ability*	0.551*	0.984*		
Lower limb strength & age & upper limb strength	0.518	1.426		
Lower limb strength & body mass index & walking ability	0.516	1.452		
Social participation				
Univariate regression				
Lower limb strength	0.35	–0.999	1.123	0.029
Walking ability	0.328	–0.768	7.137	0.043
Upper limb strength	0.297	–0.429	0.866	0.102
Grip strength	0.241	0.173	1.953	0.018
Body mass index	0.155	1.097	–0.147	0.165
Age	0.002	2.743	–0.614	0.057
Duration of tracheal intubation	0.001	2.751	–0.325	0.141
Multivariate regression (combinations of 2 variables)				
Predictor variable combination	R^2^	Cp		
Lower limb strength & walking ability	0.452	–0.105		
Lower limb strength & age	0.425	–0.072		
Multivariate regression (combinations of 3 variables)				
Predictor variable combination	R^2^	Cp		
Lower limb strength & age & body mass index	0.592	0.392		
Lower limb strength & age & walking ability	0.531	1.05		
Multivariate regression (combinations of 4 variables)				
Predictor variable combination	R^2^	Cp		
Lower limb strength & age & body mass index & duration of tracheal intubation*	0.623*	2.058*		
Lower limb strength & age & body mass index & upper limb strength	0.605	2.255		

R^2^: r-squared (higher scores are better); Cp: Mallows’ C (lower scores are better).

* Final multivariate model.

### Social participation (Reintegration of Normal Living Index)

The mean RNLI participation score (0–100) was 54.98 ± 24.97.

After univariate regression analysis, we retained our significant predictor variables in the multivariate analysis and selected the final multivariate regression model using the best subset approach (see Table III). The final multivariate regression model that explains social participation at 3 months included age, body mass index, lower limb strength, and duration of tracheal intubation (*r*^2^ = 0.6229; Mallows’ Cp = 2.0583). Therefore, younger patients with lower body mass index and a shorter duration of tracheal intubation had higher RNLI participation scores at 3 months after rehabilitation.

## DISCUSSION

Our study aimed to describe the HRQoL and participation of sepsis survivors with severe sequelae who have undergone a comprehensive and structured rehabilitation programme. Therefore, we included 100 severely affected and critically ill sepsis survivors, and assessed their outcomes 3 months after discharge from inpatient rehabilitation.

Three months after rehabilitation, 69% of our patients were living at home with or without care, indicating a substantial rate of successful community reintegration. The mean HRQoL score on the EQ-5D utility index was 0.64 ± 0.32. In comparison with a recent cohort study (13) this is a relatively moderate level of HRQoL in the study population. The mean participation score on the Reintegration of Normal Living Index (RNLI, 0–100) was 54.98 ± 24.97, and compared with another study (13) this is a relatively moderate level of social participation measured with this scale.

Our multivariate regression analyses revealed significant predictors for HRQoL and participation outcomes at 3 months. For HRQoL, age, lower limb strength, and walking ability at rehabilitation were significant predictors, explaining 55.1% of the variability in HRQoL. Regarding participation outcomes, age, body mass index (BMI), lower limb strength, and duration of tracheal intubation were significant predictors, explaining 62.3% of the variability in participation.

The study’s results suggest that even patients with severe sequelae after sepsis can achieve a reasonable health-related quality of life and social participation if managed within a specific multidisciplinary pathway including rehabilitation. Although the results seem promising at first glance, they also indicate that only 33 out of 55 patients (60%) were able to walk independently 3 months after rehabilitation. However, walking ability often has the most significant impact on personal freedom and activity compared with other physical abilities. These results suggest that patients still require therapy to achieve important goals and activities after being discharged from rehabilitation.

There might be some overlap between the 2 instruments HRQoL and RNLI. However, the predictor variables in the final regression models differ slightly. In addition, health-related quality of life and social participation are different constructs by definition. For example, the EQ5D measures mobility, self-care, usual activities, pain/discomfort, and anxiety/depression. Meanwhile, the RNLI assesses the extent to which individuals participate in their daily activities and social roles. The domains of the RNLI include indoor, community, and distance mobility, self-care, daily activities, recreational and social activities, family roles, personal relationships, presentation of self to others, and general coping skills.

A recent study, the ADRENAL trial, investigated health-related quality of life in survivors 6 months after septic shock with the EQ-5D (15). They found that about one-fifth of septic shock survivors reported moderate to extreme problems in the same HRQoL domains at 6 months as we did. They had a mean EQ-5D score of 59.4 points. Our results show that although our patients were comparable in terms of severity, measurement, and duration of illness they had slightly better health-related quality of life.

A systematic review (16) described that quality of life after critical care was worse than for age- and sex-matched populations. They also described that quality of life improved for 1 year after hospital discharge, especially physical function, physical role, vitality, and social function. Our study is in this direction: we found a moderate HRQoL 3 months after rehabilitation following sepsis.

A qualitative study showed recently that some domains emerged as critically important for people after acute sepsis, such as return to normal living (17). Our study describes for the first time that social participation, as measured by the RNLI, may be measurable for patients after severe sepsis following rehabilitation. We are not aware of any studies that investigated social participation in the short or long term after rehabilitation of critically ill sepsis survivors. We found only 2 studies that described social participation after rehabilitation of patients with CIP/CIM and/or ICU-acquired muscle weakness, conditions that many sepsis patients suffer from, 1 year after discharge from rehabilitation (13, 18). However, these studies are only partially comparable because we described a different study population with defined severe sepsis and we measured social participation earlier after rehabilitation.

In another study, HRQoL was assessed using the EQ-5D and participation in the RNLI in a patient cohort with CIP/CIM, of which 55% had consequences of sepsis (13). Six months after rehabilitation, the mean EQ-5D score was 60 points and the RNL Index score was 65 points. These findings are slightly better than in our study. However, it should be noted that these 2 study populations are not directly comparable; the patients in our study all had sepsis and were more severely affected. Nevertheless, we interpret our results as indicating moderate health-related quality of life and moderate participation. There is currently no description in the international literature of the optimal specific pathways for critically ill sepsis survivors. On our CSC pathway, patients follow a guideline-based treatment and rehabilitation pathway from the acute phase at the University Hospital Dresden to their individual rehabilitation and reintegration into normal life programme in Kreischa. It is premature to describe the effects of this pathway at this stage. However, the long-term follow-ups scheduled at 1, 2, and 3 years will determine whether patients benefit from being treated in this pathway.

### Strengths and limitations

A strength of our study is that we were able to describe certain levels of health-related quality of life (HRQoL) and participation 221 days after severe sepsis onset and at 3 months after inpatient rehabilitation. These new descriptive results provide insights into well-being and social reintegration at this stage after severe sepsis with severe sequelae.

We also describe strong improvements in the rates of weaning from mechanical ventilation, weaning from the tracheostomy tube, withdrawal from renal replacement therapy, wound healing, and walking ability in the patients who were available at the 3-month follow-up. However, due to the study design it remains unclear whether this is a result of specific rehabilitation approaches. It is important to interpret the results with caution due to the study’s limitations. The severity of the initial illness and the single-centre approach may affect the generalizability of the findings to all sepsis survivors.

The rate of complications and mortality also influences the results. On the one hand, this describes the severity of the conditions in these patients, and, on the other hand, it results in patient selection. Of the 100 patients in the initial cohort, 18 patients were transferred to other hospitals due to surgical and/or interventional procedures. Six of them had recurrent sepsis and required infectious source control through surgery, while the remaining patients had other indications such as bleeding or heart attack.

Nineteen patients died during the course of rehabilitation. At first glance, the mortality rate may seem high (around 20%). However, it is important to note that 18 of 19 patients who died at the facility had a do-not-resuscitate order as a restriction, and 11 of them additionally had a palliative care goal. Recurrent assessment of treatment goals, including the initiation of therapy limitations and palliative care concepts, is a crucial component in the management of critically ill patients and, therefore, part of our patient care pathway (19, 20). However, 8 patients were lost to follow-up after rehabilitation. Therefore, the complication rate and mortality may potentially be even higher.

Another limitation of our study is the absence of a control group. It remains unclear how much improvement could have been achieved or what the quality of life and participation of the patients would have been in another rehabilitation setting or without a multidisciplinary pathway.

It could be argued that the EQ-5D was developed without input from intensive care survivors. Its underlying domains are based on the perspective of the general population and may fail to reflect the specific experiences and concerns of sepsis survivors. However, we have had positive empirical experience with the EQ-5D in a larger cohort study of 150 patients who had acquired muscle weakness in the intensive care unit (ICU), mostly after sepsis, 1 year after being discharged from rehabilitation. The suitability of the EQ-5D in that study was excellent. Therefore, we reached a consensus to use the EQ-5D again in this sepsis project (13).

Moreover, the results of the short-term follow-up presented here might not fully reflect the long-term outcomes and challenges that patients face during their recovery. Furthermore, the results are derived from a specific cohort of critically ill patients after sepsis who underwent inpatient rehabilitation. Patients transferred to a specific rehabilitation clinic may represent a specific subset of sepsis survivors with distinct characteristics and outcomes compared with those managed in other settings or who did not receive rehabilitation. Therefore, their applicability to other patient populations and healthcare settings may differ.

Our study did not explore potential psychological and social factors that could influence HRQoL and social participation outcomes. Psychological well-being and social support are known to play significant roles in the recovery process, and their inclusion in future research could provide a more comprehensive understanding of the patients’ experiences.

To gain a more comprehensive understanding of the long-term recovery experiences and potential influencing factors, future research should include longer follow-up durations and explore additional psychosocial variables. The results from this study contribute to the growing body of evidence on the importance of rehabilitation in improving HRQoL and social participation outcomes in critically ill patients after sepsis.

### Conclusion

Patients who have experienced serious sepsis with severe sequelae can achieve a moderate level of quality of life and participation within a multidisciplinary pathway.
